# Quality of work life and associated factors among health professionals working at private and government health institutions in Awi zone, Amhara regional state, Ethiopia, 2022: a comparative cross-sectional study

**DOI:** 10.3389/fpubh.2024.1377145

**Published:** 2024-07-02

**Authors:** Agumas Fentahun Ayalew, Wei Ma, Workineh Tamir, Kefale Mitiku

**Affiliations:** ^1^Department of Epidemiology, School of Public Health, Cheeloo College of Medicine, Shandong University, Jinan, Shandong, China; ^2^Department of Public Health, College of Medicine and Health Science, Injibara University, Injibara, Ethiopia; ^3^Department of Medical Laboratory Sciences, College of Medicine and Health Science, Injibara University, Injibara, Ethiopia; ^4^Department of Biomedical Sciences, College of Medicine and Health Science, Injibara University, Injibara, Ethiopia

**Keywords:** Ethiopia, government, health professionals, private, quality of work life

## Abstract

**Background:**

Quality of work-life issues significantly impact the economic, physical, and psychological well-being of health professionals and their families. Enhancing QWL aims to foster a conducive environment and improve work performance. This study evaluated the quality of work life of health professionals in government and private health institutions in the Awi zone, Ethiopia.

**Methods:**

A comparative cross-sectional approach was employed, with study participants selected via the lottery method in 2022. Socio-demographic and organizational-related data were collected, coded, cleaned, and entered into Epi-Data version 3.1, then analyzed using SPSS version 27. Candidate variables were selected using bivariable logistic regression (*p* < 0.20). We used multivariable logistic regression to identify factors associated with quality of work life, presenting AOR with a 95% CI at a 5% significance level.

**Results:**

The study included 385 private health professionals and 395 government health professionals, with response rates of 90.38 and 92.72%, respectively. Overall quality of work-life satisfaction was 53.08% (95% CI: 49.2–57.0), with private health institution workers reporting satisfaction at 42.3% (95% CI: 37.4–47.30) and government health professionals at 63.54% (95% CI: 58.78–68.31). The difference between the two groups was 21.2% (95% CI: 14.3, 27.9). Factors significantly associated with quality of work life included type of health institutions (AOR = 2.272; 1.684, 3.065), family size (AOR = 1.536; 1.122, 2.103), personnel protective equipment (AOR = 1.369; 1.006, 1.863), eye protection (AOR = 2.090; 1.514, 2.885), engineering control (AOR = 1.563; 1.140, 2.143), and accessibility of alcohol (AOR = 1.714; 1.219, 2.410).

**Conclusion:**

Health professionals in private health institutions exhibited lower quality of work-life satisfaction than government health institutions. Quality of work life was significantly associated with the type of health institutions, family size, availability of personal protective equipment, eye protection, engineering control, and accessibility of alcohol. Regular monitoring and evaluation of the quality of work life, ensuring the availability of appropriate personal protective equipment, and providing sufficient materials and equipment for both groups were recommended based on the findings.

## 1 Introduction

Employees’ quality of work life (QWL) in different sectors can be affected in multidimensional ways. Numerous factors unique to each sector and shared challenges across industries contribute to the complexity of this impact (1). QWL consists of different dimensions used for evaluation and measurement, including work environment, organizational culture and climate, relationships and cooperation, training, compensation rewards, facilities, job satisfaction, job security, the autonomy of work, and resources (2). The QWL level of employees can affect the effectiveness and performance of the organization. Well-satisfied employees with high QWL can make their companies successful. Among the advantages of QWL are improving the quality of the work culture and experience of employees, satisfying clients’ needs, and achieving the overall objectives of the health institution (3).

The QWL for health professionals varies significantly depending on factors like country, region, urban or rural setting, and the specific challenges present in each healthcare system. However, some common themes can impact the QWL for health professionals across the continent, like inadequate budgetary allocation to health and poor leadership and management, overall workload, a lack of training and support, adverse working conditions, issues related to the local communities, and the impact of postings on nurses’ private lives. Poor working conditions and perceived lack of recognition emerged as the main demotivating factors, and some of the challenges led to decreased QWL among health professionals (4, 5). Healthcare personnel experienced seven times the national rate of musculoskeletal disorders compared with all other sector workers. It can directly affect the QWL of health professionals at and outside their workplace (6). The major influencing factors of QWL were unsuitable working hours, a lack of facilities for nurses, the inability to balance work with family needs, the inadequacy of vacation time for nurses and their families, poor staffing, management, and supervision practices, a lack of professional development opportunities, and an inappropriate working environment in terms of the level of security, patient care supplies and equipment, and recreation facilities (break area) (7). And the perception of nurses in the community and low pay are two additional crucial elements. Similarly, gender, age, marital status, family size, nursing tenure, organizational tenure, positional tenure, and payment per month were the influential factors for QWL (7).

**Figure 1:**
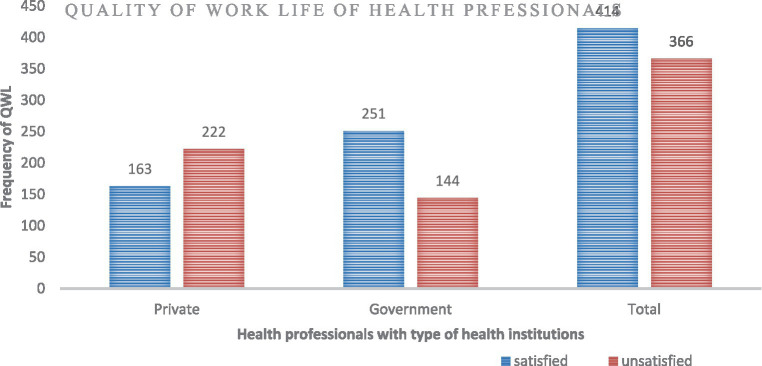
Quality of life of health professionals working at private and government health institutions in Awi zone, Amara regional state, Ethiopia, 2022 (*n* = 780).

Ethiopian HPs face various challenges. These challenges may affect several aspects of their work and personal lives. For example, limited resources, workload, and staffing issues, geographical disparities, compensation and incentives, training and professional development, inadequate housing and amenities, ethical challenges, disease burden, political and economic instability, and workplace safety are the challenges of most HPs, which can affect the quality of health service provision and the safety of HPs in general (8, 9). Addressing those challenges requires such studies to increase the visibility of the problems and involve a combination of government policies, investments in healthcare infrastructure, workforce planning, and initiatives to improve education and training for HPs. Knowing the QWL is crucial for organizations to develop and train productive and efficient employees to fulfill their job tasks. Thus, this study was performed to establish the available data on HPs’ QWL, the availability of qualified supervisors in QWL, and the measure of QWL in HIs. Information on the availability of PPE, chemical disinfectants or antiseptics, and the frequency of antiseptic or sanitizer utilization in HIs was also sought at government and private health institutions in Awi zone, Amhara regional state, Ethiopia, in 2022 (Figure 1).

## 2 Materials and methods

### 2.1 Study area, design, and period

We used private and government health institutions in the Awi Zone as a study area. Awi Zone is one of the 15 zones found in the Amhara Regional State. This zone includes nine woredas, three city administrations, and 233 kebeles. According to the zonal administration office, the current population of this zone exceeds 1,230,000. Injibara is the capital city of Awi Zone, located 445 km (about 276.51 mi) from Addis Ababa, the capital city of Ethiopia, and 122 km (about 75.81 mi) from Bahir Dar, the capital city of the Amhara Regional State. The study area encompasses one general hospital, four primary hospitals, and 46 health centers. According to the Awi Zone health office, there are 2,134 health professionals in these 46 government health centers. This zone has three specialized, 44 medium, and 134 primary private clinics. Additionally, there were 19 pharmacies, 118 drug stores, and three drug vendors in the zone. A total of 720 health professionals work in these private health institutions. An institutional-based comparative cross-sectional study was conducted from November 1 to December 30, 2022.

### 2.2 Study population, inclusion, and exclusion criteria

The study population consisted of all health professionals working in private and government health institutions in the study area, who asked for their permission to participate without restriction on their profession. Inclusion criteria included health professionals with incredible work experience of 6 months or longer. However, health professionals on annual leave, sick leave, maternal leave, and those out of health institutions for more than the data collection period due to other factors were excluded from the study. Furthermore, health professionals from hospitals were also excluded from the study due to the higher standards typically found in governmental hospitals, while there were no private hospitals in the study area during the study period.

### 2.3 Sample size determination

The sample size was calculated using Epi Info 7 software STATCALC and the double population proportion formula: by considering two groups have a ratio of equal sample size (1:1), with the assumptions of a 95% confidence level, power of 80, 5% of marginal error and proportion of QWL for private health institutions professionals were P_1_ = 50% since there was no previous study conducted in the area. This 50% proportion would provide the maximum sample size and variance (10). Similarly, there were no previous studies on the QWL of government health professionals. Therefore, a 10% difference of was used between the two groups, hence we took P_2_ = 60% as the proportion of QWL of government health institution professionals. Based on these assumptions, the required sample size was n_1_ = 386.90 = 387; n_2_ = 1×387 = 387; and we took approximately 774 study participants from both groups. The final smallest required sample size was assumed to be the calculated sample size plus a 10% non-response rate; so, the final smallest required sample size was 852 (426 from private health institutions and 426 from government health institutions).

### 2.4 Sampling procedures

First, we defined the population as the study involved all health professionals working in governmental and private health institutions in the Awi Zone. We determined the total number of health professionals in the study area for both groups, which were 2,134 for government and 720 for private health institutions. Then, we allocated the required sample size proportionally according to the number of health professionals in each health institution. A unique identifier was assigned to each health professional based on employee ID numbers within the study group and according to their institution. The lottery process was executed at each health institution level using these unique identifiers to select the participants. Data collectors formally communicated with the selected health professionals, using their unique identifiers to notify them about their participation.

### 2.5 Study variables, data collection tool, and procedures

The dependent variable was QWL, categorized as satisfied and unsatisfied, while independent variables included socio-demographic characteristics (age, sex, educational level, religion, work experience, department or profession, monthly income, marital status), organizational factors (type of health institution, PPE, engineering and administrative control, working hours, training), collected using structured questionnaires through direct observation and interview methods. The questionnaire was adopted and modified from earlier comparable studies (2), prepared in English, and did not need translation into the local language, as the study participants (health professionals) could understand English. Twelve health professionals were selected for data collection and supervision, with nine for data collection and three for supervision.

### 2.6 Data quality assurance

Two days of training were provided on the study’s objective, the questionnaire contents, confidentiality, respondents’ rights, and data collection procedures to data collectors and supervisors. A pre-test was conducted on 5% of the sample (*n* = 42), and the tool was modified for inconsistencies and ambiguity before actual data collection.

### 2.7 Operational definition

Quality of work life is an employee’s self-reported satisfaction with a job. This is usually interpreted to include how well the job contributes to their overall quality of life. It refers to an employee’s satisfaction with the working life and emphasizes the quality of the relationship between the worker and the working environment (11). QWL was assessed based on an individual’s life, considering that disturbances in personal life can affect professional life and vice versa. Employees were categorized as satisfied or unsatisfied based on their QWL score. First, participants’ responses were computed from various nine components such as work environment, organizational culture and climate, relationships and cooperation, training and development, compensation and rewards, facilities, the autonomy of the work, and adequate resources contributed to the overall QWL score. Then, the computed scores were divided by the total number of QWL questionnaires 50. Finally, the mean was computed and used to describe the level of satisfaction as satisfied and unsatisfied. Individuals with a score greater than or equal to the overall mean were categorized as satisfied, while those with a score less than the overall mean were categorized as unsatisfied (2).

### 2.8 Data processing and analysis

After data collection, it was checked for completeness, coded, entered into the Epi-Data version 3.1, and transferred to SPSS version 27 for cleaning and analysis. Descriptive statistics, including tables, graphs, pie charts, and proportions, were used for data presentation. Bivariate and multivariate logistic regression analyses were performed to determine the correlation between dependent and independent variables. During the model-building process, diagnostic checks were conducted to assess the availability of multicollinearity between variables using variance inflation factors and all variables had VIF values less than 10. The Hosmer and Lemeshow goodness-of-fit tests were used to check assumptions and model fitness to the observed data, and it was fulfilled at X^2^ = 12.830 and *p* = 0.118 since it was >0.05. A *p* < 0.20 was used as the cut-off for bivariate logistic regression, while a *p* <0.05 was used for multivariable logistic regression.

## 3 Results

### 3.1 Socio-demographic characteristics of the respondents

A total of 780,385 private and 395 government HPs participated in the study, with 90.4 and 92.7% response rates for private and government HPs, respectively. Diploma-level HPs account for 65.7% (*n* = 253) of private HIs and 56.5% (*n* = 223) of government HIs. Most private HPs have less than 5,000 birrs in monthly income, whereas most government HPs have between 5,000 and 10,000 birrs. Of the participants, 57.8% (*n* = 451) were males and 42.2% (*n* = 329) were females. In terms of professional departments, 51.9% (*n* = 200) were from private HIs, 52.7% (*n* = 208) were from government HIs, and 52.3% (*n* = 408) of the total participants were nurses (Table 1).

**Table 1:** Socio-demographic characteristics of health professionals at private and government health institutions in Awi zone, Amhara regional state, Ethiopia, 2022 (*n* = 780).

Characteristics		Health professionals with health institution types			
		Private health institution (*n* = 385)	Government health institution (*n* = 395)	Total (*n* = 780)	
Variables	Category	Frequency (%)	Frequency (%)	Frequency (%)	Χ^2^, *p*-value
Age	< 29	243(63.1)	250(63.3)	493(63.2)	Χ^2^ = 6.5, *p* = 0.090
	30–39	117(30.4)	110(27.8)	227(29.1)	
	40–49	20(5.2)	29(7.3)	49(6.3)	
	≥ 50	5(1.3)	6(1.5)	11(1.4)	
Sex	Male	218(56.6)	233(59.0)	451(57.8)	Χ^2^ = 0.2, *p* = 0.624
	Female	167(43.4)	162(41.0)	329(42.2)	
Educational level	Diploma (< Bachler)	253(65.7)	223(Χ^2^56.5)	476(61.0)	Χ^2^ = 0.3, *p* = 0.592
	≥ Degree (Bachler’s)	132(34.3)	172(43.5)	304(39.0)	
Work experience	<5 years	248(64.4)	234(59.2)	482(61.8)	Χ^2^ = 8.9, *p* = 0.012
	5–10 years	98(25.5)	102(25.8)	200(25.6)	
	>10 Years	39(10.1)	59(14.9)	98(12.6)	
Monthly income	<5,000	287(74.5)	184(46.6)	471(60.4)	Χ^2^ = 0.9, *P* = 0.624
	5,000–10,000	98(25.5)	203(51.4)	301(38.6)	
	>10,000	0(0.0)	8(2.0)	8(1.0)	
Department	Pharmacy	98(25.5)	50(12.7)	148(19.0)	Χ^2^ = 6.6, *p* = 0.160
	Nurse	200(51.9)	208(52.7)	408(52.3)	
	Laboratory	70(18.2)	49(12.4)	119(15.3)	
	Midwifery	0(0.0)	48(12.2)	48(6.2)	
	Others (HO, HIT, MD)	17(4.4)	40(10.1)	57(7.3)	
Marital Status	Single	200(51.9)	193(48.9)	393(50.4)	Χ^2^ = 7.1, *p* = 0.028
	Married	175(45.5)	189(47.8)	364(46.7)	
	Others	10(2.6)	13(3.3)	23(2.9)	
Family size	1	198(51.4)	205(51.9)	403(51.7)	Χ^2^ = 10.0, *p* = 0.075
	2	50(13)	38(9.6)	88(11.3)	
	≥ 3	137(35.6)	152(38.5)	289(37.1)	
Working at d/t His	Yes	200(51.9)	167(42.3)	367(47.1)	Χ^2^ = 6.1, *p* = 0.013
	No	185(49.1)	228(57.7)	413(52.9)	

HO, health officer; HIT, health information technician; MD, medical doctor.

### 3.2 Availability of personal protective equipment

Regarding the availability of PPE, 60% (*n* = 231) of private HPs and 48.9% (*n* = 193) of governmental HPs had a shortage of gloves in their rooms. About one-fourth, 28.6% (*n* = 110), of private HPs had eye or face protection (e.g., goggles, a face shield) used during service provision. Meanwhile, one-third, 35.7% (*n* = 141), of governmental HPs had eye or face protection and used it during service provision (Table 2).

**Table 2:** Availability of personnel protective equipment among health professionals at private and government health institutions in Awi zone, Amhara regional state, Ethiopia, 2022 (*n* = 780).

Characteristics		Health professionals with health institution types			
		Private health institution workers (*n* = 385)	Governmental health institution workers (*n* = 395)	Total (*n* = 780)	
Variables	Category	Frequency (%)	Frequency (%)	Frequency (%)	Χ^2^, *p*-value
Do you have appropriate personnel protective equipment?	No	176(45.7)	167(42.3)	343(44.0)	Χ^2^ = 12.1, *p* = 0.001
	Yes	209(54.3)	228(57.7)	437(56.0)	
Do you have a face mask to wear while serving the client?	No	128(33.2)	101(25.6)	229(29.4)	Χ^2^ = 4.6, *p* = 0.033
	Yes	257(66.8)	294(74.4)	551(70.6)	
Do you have enough gloves in your room?	No	231(60.0)	193(48.9)	424 (54.4)	Χ^2^ = 3.0, *p* = 0.083
	Yes	154(40.0)	202(51.1)	356(45.6)	
Do you have eye or face protection (e.g., goggles or a face shield)?	No	275(71.4)	254(64.3)	529(67.8)	Χ^2^ = 25.3, *p* < 0.001
	Yes	110(28.6)	141(35.7)	251(32.2)	
Do you have disinfectants around your working area?	No	147(38.2)	157(39.7)	304(39.0)	Χ^2^ = 0.1, *p* = 0.958
	Yes	238(61.8)	238(60.3)	476(61.0)	
Do you have antiseptics around your working area?	No	134(34.8)	142(35.9)	276(35.4)	Χ^2^ = 11.4, *P* = 0.001
	Yes	251(65.2)	253(64.1)	504(64.6)	
Do you have an accessible hand-washing facility in your working room?	No	142(36.9)	99(25.1)	241(30.9)	Χ^2^ = 1.9, *p* = 0.166
	Yes	243(63.1)	296(74.9)	539(69.1)	
Do you have soap or bleach in your room?	No	168(43.6)	177(44.8)	345(44.2)	Χ^2^ = 12.1, *P* < 0.001
	Yes	217(56.4)	218(55.2)	435(55.8)	
Total	No	199(51.69)	185(46.84)	384(49.23)	Χ^2^ = 25.0, *P* < 0.001
	Yes	186(48.31)	210(53.17)	396(50.77)	

### 3.3 Engineering and administrative control

Most participants, 63.8% (*n* = 498), did not restrict the number of personnel entering the patients’ rooms. From this variable, private HPs took the lion’s share, 68.8% (*n* = 265), and government HPs accounted for 59% (*n* = 233). When we assess the availability of facilities, a policy includes notifying us if there are clusters of respiratory illnesses. 50.9% (*n* = 196) of private HPs and 41.5% (*n* = 162) of government HPs respond that they have no facility policy that includes notifying if there are clusters of respiratory illnesses (Table 3).

**Table 3:** Engineering and administrative control among health professionals working at private and government health institutions in Awi zone, Amhara regional state, Ethiopia, 2022 (*n* = 780).

Characteristics		Health professionals with health institution types			
		Private health institution workers (*n* = 385)	Government health institution workers (*n* = 395)	Total (*n* = 780)	
Variables	Category	Frequency (%)	Frequency (%)	Frequency (%)	Χ^2^, *p*-value
Restrict the number of personnel entering a patient’s room.	No	209(54.3)	235(59.5)	444(56.9)	Χ^2^ = 0.3, *p* = 0.596
	Yes	176(45.7)	160(40.5)	336(43.1)	
Minimize the number of staff present when performing aerosol-generating procedures.	No	231(60.0)	240(60.8)	471(60.4)	Χ^2^ = 7.0, *p* = 0.008
	Yes	154(40.0)	155(39.2)	309(39.6)	
It is possible to isolate suspected cases to help prevent transmission.	No	150(39.0)	185(46.8)	335(42.9)	Χ^2^ = 2.9, *p* = 0.087
	Yes	235(61.0)	210(53.2)	445(57.1)	
Engineering controls to shield healthcare workers from patients	No	265(68.8)	233(59.0)	498(63.8)	Χ^2^ = 19.2, *p* < 0.001
	Yes	120(31.2)	162(41.0)	282(36.2)	
Are there IPC program standards and policies in your organization?	No	141(36.6)	159(40.3)	300(38.5)	Χ^2^ = 1.5, *p* = 0.225
	Yes	244(63.4)	236(59.7)	480(61.5)	
Does the facility’s policy include notifying people if there are clusters of respiratory illnesses?	Yes	196(50.9)	164(41.5)	360(46.2)	Χ^2^ = 10.1, *P* = 0.001
	No	189(49.1)	231(58.5)	420(53.8)	
Use an alcohol-based hand rub after you serve each client.	No	103(26.8)	93(23.5)	201(25.8)	Χ^2^ = 13.9, *P* < 0.001
	Yes	282(73.2)	302(76.5)	579(74.2)	

### 3.4 Quality of work life, dimensions, and comparisons among private and government health professionals

The quality of work-life scale reliability analysis results of Cronbach’s alpha in this study was equal to 0.952. This scale consisted of 50 items and demonstrated high internal consistency. Using this measuring tool, less than half of private HPs were satisfied with their QWL: 42.3% (*n* = 163) (95% CI: 37.4–47.30), whereas 63.54 (*n* = 251) (95% CI: 58.78–68.31) of government HPs were satisfied with their QWL. Meanwhile, the overall QWL satisfaction of health professionals was 53.08% (*n* = 414) (95% CI: 49.2–57.0) (Figure 1). This indicated that the QWL was lower among private health institution workers than government health institution workers, with a point estimate for the difference of 21.20% (95% CI: 14.30, 27.90). Regarding the dimensions of QWL, according to this study, governmental health professionals had better satisfaction in all dimensions of the QWL components than working professionals in private health institutions. There were statistically significant differences between health institution types across all nine dimensions at *p* < 0.05. The greatest difference between the two study groups was at the facilities dimension of 17.9% (95% CI: 10.9, 24.7), whereas the smallest difference was in the adequacy of the resources dimension of 8.4% (95% CI: 1.5, 15.2) (Table 4).

**Table 4:** QWL and its dimensions comparisons and independent sample proportions test results among private and government health professionals Awi zone, Amhara regional state, Ethiopia, 2022 (*n* = 780).

Variables		Types of HIs	Frequency	Success	Proportion	Proportion deference	*P*-value	[95% CI of difference]
QWL		Private	385	163	0.423	0.212	*P* < 0.001	[0.143, 0.279]
		Government	395	251	0.635			
Dimensions of quality of work life	Work environment	Private	385	184	0.478	0.137	<0.001	[0.068, 0.206]
		Government	395	243	0.615			
	Organizational culture and climate	Private	385	198	0.514	0.119	<0.001	[0.049, 0.187]
		Government	395	250	0.633			
	Relationships and cooperation	Private	385	196	0.509	0.091	0.011	[0.021, 0.160]
		Government	395	237	0.600			
	Training and development	Private	385	161	0.418	0.141	<0.001	[0.071, 0.210]
		Government	395	221	0.559			
	Compensation and rewards	Private	385	175	0.455	0.158	<0.001	[0.088, 0.226]
		Government	395	242	0.613			
	Facilities	Private	385	169	0.439	0.179	<0.001	[0.109, 0.247]
		Government	395	244	0.618			
	Job satisfaction and job security	Private	385	188	0.488	0.150	<0.001	[0.080, 0.218]
		Government	395	252	0.638			
	Autonomy of work	Private	385	164	0.426	0.169	<0.001	[0.099, 0.237]
		Government	395	235	0.595			
	Adequacy of resources	Private	385	148	0.384	0.084	0.018	[0.015, 0.152]
		Government	395	185	0.468			

QWL, quality of work life; HI, health institution; CI, confidence interval.

### 3.5 Factors associated with QWL among private and governmental health professionals

Types of health institutions, family size, availability of appropriate PPE, availability of eye protective material, and accessibility of alcohol-based hand rub had statistically significant associations with quality of work life among private and governmental health professionals at a *p* < 0.05 during multivariable logistic regression. Government HPs were 2.3 times more likely to be satisfied with their QWL than private HPs (AOR = 2.272; 1.684, 3.065). Those HPs with only one family size were 1.6 times more likely to satisfy their QWL than HPs with ≥ three family sizes (AOR = 1.581; 1.147, 2.180). Health professionals with eye protective material were 2.0 times more likely to be satisfied with their quality of life than their counterparts (AOR = 1.968; 1.414, 2.741). Health professionals with engineering control at their institution were 1.4 times more likely to be satisfied than those without (AOR = 1.393; 1.003; 1.936). Similarly, health professionals who had access to alcohol for hand rub after service were nearly 1.7 times more likely to be satisfied with their QWL than their counterparts (AOR = 1.690; 1.193, 2.392), and HPs who had appropriate PPE were 1.4 times more likely to be satisfied with their QWL than those who did not have appropriate PPE (AOR = 1.369; 1.006, 1.863) (Table 5).

**Table 5:** Bi-variable and multi-variable analysis of quality of life of health professionals working at private and government health institutions in Awi zone, Amara regional state, Ethiopia, 2022 (*n* = 780).

Variables	Category	Quality of work life		COR (95%CI)	AOR (95%CI)
		Unsatisfied	Satisfied		
Health institutions	Government	144	251	2.374(3.780,3.166)***	2.272(1.684,3.065)***
	Private	222	163	1	1
Family size	1	169	234	1.558(1.149, 2.111)**	1.581(1.147,2.180)**
	2	44	44	1.125(0.698, 1.813)	1.301(0.787,2.153)
	≥3	153	136	1	1
Availability of eye protection	No	281	248	1	1
	Yes	85	166	2.213(1.619,3.024)***	1.968(1.414,2.741)***
Availability of engineering control	No	263	235	1	1
	Yes	103	179	2.610(1.654,4.120)***	1.393(1.003,1.936)*
Availability of alcohol-based hand rubs	No	117	84	1	1
	Yes	249	330	1.846(1.334,2.555)***	1.690(1.193,2.392)**
Availability of appropriate PPE	No	185	158	1	1
	Yes	181	256	1.656(1.245,2.202)**	1.369(1.006,1.863)*

*Statistically significant at *P* < 0.05, ** < 0.01, *** < 0.001.

## 4 Discussion

The findings of this study shed light on the significant disparities in QWL between private and governmental HPs, offering insights into potential predictors and implications for healthcare institutions and policy. While acknowledging the nuances and limitations inherent in cross-sectional studies, the robustness of the associations identified underscores the importance of addressing key factors influencing HPs’ QWL for organizational effectiveness and healthcare outcomes. In this study, only 42.3% (*n* = 163) (95% CI: 37.4–47.3) of private HPs were satisfied with their QWL. While about 63.5% (*n* = 251) (95% CI: 58.78–68.31) of governmental HPs were satisfied with their QWL, the overall satisfaction with QWL was 53.1% (*n* = 414) (95% CI: 49.2–57.0). QWL was lower among private HPs, with a point estimate for the difference of 21.2% (95% CI: 14.3, 27.9). The greatest difference between the two study groups came from the facilities dimension of 17.9% (95% CI: 10.9, 24.7), whereas the smallest difference was in the adequacy of the resources dimension of 8.4% (95% CI: 1.5, 15.2). Although the smallest difference was shown in this dimension, it was the worst dimension in both study groups; only 38.4 and 46.8% of participants were satisfied with the adequacy of resources from private HPs and government HPs, respectively. This suggests that the shortage of PPE influences private HIs workers, such as lack of eye protection, lack of engineering control at their organization, and unavailability of alcohol-based hand rub, which, consequently, affects their QWL. The observed discrepancy in QWL between private and governmental HPs reflects broader systemic challenges within the healthcare sector, ranging from resource allocation to organizational culture. The lower satisfaction among private HPs highlights critical areas of concern, particularly regarding inadequate PPE, suboptimal working conditions, and limited professional development opportunities. These findings underscore the pressing need for targeted interventions to enhance the QWL of private HPs, thereby improving overall healthcare delivery and outcomes. The overall QWL of HPs was in line with the study conducted in Arab countries after the COVID-19 pandemic (12). Moreover, it was higher than the study conducted in Greece (13). This might be due to this study being conducted among HPs working with refugees and migrants. However, the overall QWL was much lower than in another study conducted in Saudi Arabia (14). These might be due to better monthly income and working hours for Saudi Arabian HPs.

In addition to assessing the QWL, this study also predicts various predictors of QWL. Multiple factors can influence the QWL for HPs, and health institution type is among those factors that can play a significant role (AOR = 2.272, 1.684, 3.065). This implies that numerous problems, such as low monthly income, inadequate PPE, lack of compensation, inappropriate working conditions, job insecurity, inadequate professional development, and issues about personal preferences, hurt the quality of work life for private employees (15). The study revealed that increasing family size made HPs more likely to be unsatisfied regarding their QWL (AOR = 1.581; 1.147, 2.180). This might be due to the increased workload and financial requirements, while there needs to be more exceptional support from the institutional side. The availability of appropriate PPE (AOR = 1.369; 1.006, 1.863) and eye protection material (AOR = 1.968; 1.414, 2.741) were among the predictors of QWL. Health professionals with appropriate PPE and eye protection material were more likely to be satisfied with their QWL than their counterparts. Appropriate PPE and eye-protective materials play a crucial role in safeguarding the health of HPs; otherwise, if there is a lack of proper PPE, they might be exposed to infectious agents or other hazards (16, 17). Therefore, the use of PPE prevents the transmission of diseases, reduces the risk of occupational exposures, maintains the well-being of healthcare workers, and leads to the advancement of the QWL of HPs. The quality of work life among health professionals with engineering control at their organization was 1.4 times higher than those without engineering (AOR = 1.393, 1.003, 1.936). This also might affect concerns about workplace safety, including exposure to infectious diseases, physical hazards, and violence, which can directly impact HPs’ well-being and lead to decreased QWL. Health professionals who practice alcohol-based hand rub were 1.7 times more likely to be satisfied with their QWL than their counterparts (AOR = 1.690; 1.193, 2.392). This is also linked to safety concerns, including exposure to infectious diseases and a lack of proper safety measures, which can impact HPs’ physical and mental well-being. The identified predictors of QWL offer valuable insights into potential areas for intervention and improvement. Factors such as family size and access to appropriate PPE emerge as significant determinants, emphasizing the complex interplay between personal and organizational factors in shaping HPs’ work experiences. Moreover, the association between engineering controls and QWL underscores the pivotal role of workplace safety in fostering a conducive environment for HPs.

In general, the findings underscore the critical importance of prioritizing HPs’ QWL as a cornerstone of effective healthcare delivery. By addressing key determinants such as access to adequate resources, workplace safety measures, and professional development opportunities, healthcare institutions can foster a supportive environment conducive to employee satisfaction and well-being. Ultimately, investing in the QWL of HPs not only enhances organizational performance but also contributes to improved patient outcomes and overall healthcare quality. To improve health professionals’ performance, health institutions must create suitable working conditions that offer appropriate incentives for employees to satisfy them with the system running in the institution. The inability to address the above issues can negatively impact employee behavioral responses, such as job satisfaction, job performance, turnover intention, executive turnover, and personal alienation and the outcomes of the organization (18). Therefore, this study’s findings might increase the quality of human resources in health institutions. A health professional with a happy QWL is productive, dedicated, and committed. If HPs in a particular HI have a high QWL, they might attract new employees and retain a permanent workforce (19). They have a high commitment to the HIs. High professional commitment might contribute to the health system by increasing the quality of service for both clients and HIs. It is also used to systematically manage the QWL, addressing regulatory, technical, organizational, and managerial aspects vital to attaining safer and healthier workplaces. Additionally, it would be the key to improving the patients’ and clients’ safety at HIs. This study reduced the spread of infectious diseases and other work hazards. Healthcare planners would utilize the information generated from the study to improve health service quality.

Even if this study was the first and single study that investigated and compared QWL of HPs at private HIs and government HIs within a single study in the study area, it has different limitations like other cross-sectional studies. First, the cross-sectional nature of the studies did not establish causal relationships but instead allowed for the identification of associations between the quality of work life and numerous factors among health professionals. Second, since the study used snapshot data and self-reported questionnaires, which may be exposed to recall and other biases, it may not provide a longitudinal perspective on how the QWL may have changed over time within each setting. Third, our study did not assess some factors such as building infrastructure or disabilities among health professionals. While we focused on individual and organizational factors influencing QWL. Other limitation of this study was the sample size calculated by considering 50% proportions for the first population and assuming a 10% proportion difference from the second population without direct reference to proportions reported in previous studies. Even though calculating sample size by using a 50% proportion would provide the maximum sample size and variance, the absence of specific references to prior research may introduce uncertainty regarding the generalizability of our findings. Therefore, further research is recommended for researchers to consider addressing this aspect in future work.

## 5 Conclusion

Private HPs were less satisfied with their QWL than government HPs. Types of HIs, family size, availability of appropriate PPE, availability of face protection, availability of engineering control, and accessibility of alcohol for hand rub had statistically significant associations with the QWL of HPs.

## Data availability statement

The raw data supporting the conclusions of this article will be made available by the authors, without undue reservation.

## Ethics statement

The studies involving humans were approved by Injibara University, College of Medicine and Health Science. The studies were conducted in accordance with the local legislation and institutional requirements. The participants provided their written informed consent to participate in this study.

## Author contributions

AFA: Conceptualization, Data curation, Formal analysis, Funding acquisition, Investigation, Methodology, Project administration, Resources, Software, Supervision, Validation, Visualization, Writing – original draft, Writing – review & editing. WM: Conceptualization, Formal analysis, Investigation, Methodology, Software, Supervision, Validation, Visualization, Writing – review & editing. WT: Data curation, Funding acquisition, Methodology, Resources, Writing – original draft. KM: Data curation, Funding acquisition, Investigation, Methodology, Resources, Writing – original draft.
